# Reflections on the theory of "silver bullet" octreotide tracers: implications for ligand-receptor interactions in the age of peptides, heterodimers, receptor mosaics, truncated receptors, and multifractal analysis

**DOI:** 10.1186/2191-219X-1-9

**Published:** 2011-07-26

**Authors:** Roy Moncayo

**Affiliations:** 1 Department of Nuclear Medicine, Medical University of Innsbruck, Innsbruck, Austria; 2 WOMED, Karl-Kapferer-Strasse 5, 6020 Innsbruck, Austria

**Keywords:** Michaelis-Menten, ligand, receptor, GPCR, somatostatin, octreotide, homodimers, heterodimers, receptor mosaics, multifractal analysis, morphogens, morphostats

## Abstract

The classical attitude of Nuclear Medicine practitioners on matters of peptide-receptor interactions has maintained an intrinsic monogamic character since many years. New advances in the field of biochemistry and even in clinical Nuclear Medicine have challenged this type of thinking, which prompted me to work on this review. The central issue of this paper will be the use of somatostatin analogs, i.e., octreotide, in clinical imaging procedures as well as in relation to neuroendocirne tumors. Newly described characteristics of G-protein coupled receptors such as the formation of receptor mosaics will be discussed. A small section will enumerate the regulatory processes found in the cell membrane. Possible new interpretations, other than tumor detection, based on imaging procedures with somatostatin analogs will be presented. The readers will be taken to situations such as inflammation, nociception, mechanosensing, chemosensing, fibrosis, taste, and vascularity where somatostatin is involved. Thyroid-associated orbitopathy will be used as a model for the development of multi-agent therapeutics. The final graphical summary depicts the multifactorial properties of ligand binding.

## The setting

In past issues of the *European Journal of Nuclear Medicine and Molecular Imaging*, some articles have pointed out puzzling aspects concerning ligand-receptor interactions. Rolleman et al. have documented the situation of an apparent positive cooperation between non-labeled somatostatin (SST) analogs and a radio-labeled compound *in vivo *[1]. A similar situation of increased tracer binding in the presence of 100 μg of cold octreotide had been shown earlier by Hofland [2]. These data seem to contradict some views of ligand-receptor interactions which constitute the basis of the biochemical and pharmaceutical work that is daily applied in Nuclear Medicine imaging.

The aim of this short review is to assemble recently available information on the physiological roles of somatostatin and similar substances, on modern concepts on receptors, and on binding modulators, in order to attempt to arrive at a new level of interpretation that will put a new light on scintigraphic and binding data. These data should also be a guiding complement for new peptide tracers being developed [3].

### Introduction: the basics of receptor binding and the use of octreotide

The concepts regarding receptor function have been accommodated over time to a reductionist model that ideally considers one ligand and one receptor. The basic theories behind were developed between 1900 and 1920 [4-7]. In 1956, the concept of the ability of a drug to induce an effect after binding-efficacy-was introduced by Stephenson [8]. This way of thinking fits into the metaphor of the "silver bullet", i.e., a straightforward solution thought to have outmost effectiveness (or efficacy). Based on the theories of an allosteric receptor model, Thron discussed in 1973 the interplay between agonists and antagonists [9]. These theorems have found acceptance in the field of Nuclear Medicine [10] and have been the basis for experimental and clinical work extending into the modern field of peptide therapy using SST analogs (SSA) such as octreotide and lanreotide.

The most known theorem regarding ligand interactions is the Michaelis and Menten reaction [6], defined as *v *= (*V*max^.^*S*)/(*S *+ *K*_m_). This equation has been revised recently from the stand point of fractal kinetics [11] in order to attempt to reach a higher level of understanding of the biochemical reactions found intracellularly [12]. Aranda et al. state: "Classical enzyme kinetics, which assumes the Michaelis-Menten paradigm with perfectly mixed reactants and homogeneous media, is strongly limited for applications including intracellular enzyme reactions. A major difference between a diluted enzymatic system and that found inside the cell is the high mechanical and rheological complexity of the cytoplasmic environment that produces anomalous diffusion phenomena seriously affecting enzyme kinetics of biochemical pathways" [13]. By a simple process of logical deduction, we should then expect to have *in vivo *a highly complex whole body situation when different types of tissues are being examined through scintigraphy with octreotide or other tracers in general.

The basis for the development of SST receptor (SSTR) imaging can be traced back to the research work done by Roger Guillemin [14,15]. Somatostatin was first described in 1973 by Brazeau et al. [16]. The same communication reported the bioactivity of a synthetic replicate. From the industrial point of view, researchers advanced the development of analogs quite soon after the discovery of somatostatin. The initial work was based on peptide chemistry by which the SST sequences related to peptide binding were identified [17-19]. In order to validate the binding ability, ligand binding assays were established [20]. On clinical grounds, one of the first applications of unlabelled octreotide was the treatment of acromegaly [21]. In the field of Nuclear Medicine, radioactive-labeled octreotide tracers have been in clinical use since the 1990s [22,23] becoming an established diagnostic procedure [24]. The characteristics of natural and synthetic analogs in relation to receptor internalization, as well as the conformational changes due to labeling with Yttrium or Gallium have been recently summarized by Hofland and Lamberts [25], and by Deshmukh et al. [26], respectively.

It has to be mentioned that some of the *in vitro *research with SSA in relation to neuroendocrine tumors (NET) is based on the use of pancreatic carcinoma cell lines which had been chemically induced by azaserine [27]. The histological picture of these tumors varies from "poorly differentiated solid carcinomas to well-differentiated variants which form acini" (rat CA20948 tumors) [27]. Due to the diversity of forms of NET, one should be cautious when extrapolating results from these *in vitro *observations [28]. Unfortunately this cell line is still in use in 2011.

At the present time, a high level of technological development has been reached by combining Ga^68^-labeled SST analogs and whole body PET-CT scanning [29] which now delivers more information about tracer distribution. In view of this advanced imaging situation with a higher level of molecular resolution, it is important to review the physiology related to SSTR imaging in order to come to an adequate interpretation.

#### Somatostatin receptors in fibrosis, vascularity, inflammation, and taste

Since the 1990s, one main application of SSTR imaging is in the diagnosis of neuroendocrine tumors [23]. At the time these tumors are diagnosed, there is usually clinically evident hepatic involvement. One yet unexplained biological characteristic of carcinoids is that to tend to develop fibrosis [30,31]. It should be kept in mind that liver fibrosis is accompanied by SSTR expression [32]. The role of stromal fibrosis in connection with octreotide uptake has been described by Öhrwall et al. [33]. Lebtahi et al. have shown the influence of pulmonary tissue fibrosis on octreotide uptake [34]. It follows that scintigraphic evidence of octreotide uptake might be an expression of fibrosis, which unfortunately cannot be distinguished from tumor. In a disease characterized by fibrosis, i.e., fibrous dysplasia, Chen et al. documented octreotide uptake which remained unchanged even after treatment with Sandostatin^® ^[35]. Besides fibrosis, another cause for apparent increase of binding sites in a tumor might be in relation with blood vessels [36]. This same property has been praised as a therapeutical option of somatostatin, i.e., that of being anti-angiogenic [37,38].

Experimental studies in macaques conducted by Guo et al. [39] have demonstrated the pattern of physiological development and expression of SST in the intestinal tract. The animals showed expression in mucosal crypts and as well as in the myenteric nerve plexus. Concomitant to this development the concentration of SST in the liver declined. Deficiency of SSTR2 can alter the mesenteric sensitivity of afferent nerves upon distention, i.e., mechanosensing, or acid exposure, i.e., chemosensing [40]. In addition, SST containing neurons can be found in the enteric mucosa [41]. Under experimental fasting conditions, the number of SSTR can diminish and return to normal after refeeding [42].

Recently Gonkowski and Całka [43] have demonstrated a modulation of SST immunoreactivity in the nervous structures of the porcine descending colon under experimental pathological conditions. In situations of intestinal inflammation, one can find changes in the concentration of SST as well as of SSTR [44]. Intestinal inflammation can be worsened when SSTR are not present [45]. In other words, these findings imply that a functioning SST system seems to be important in the control of intestinal inflammation [46].

In view of these data, we should ask ourselves: is there a link between intestinal inflammation and carcinoids? Recent studies have indeed delivered evidence relating both processes. West et al. have shown that carcinoids are 15 times more common in patients with Crohn's disease [47]. In a similar way, Grassia et al. have analyzed the setting of ulcerative colitis [48] and proposed that long-standing inflammation could induce changes towards tumor development. Another interesting link between inflammation and carcinoids has been provided by Sciola et al. [49]. The authors documented that high levels of chromogranin A, a marker of neuroendocrine tumors, can occur in patients with inflammatory bowel disease. In a more general way, one has to consider the interactions of the enteric nervous system with the immune system [50] and situations of intestinal inflammation [51,52]. In an attempt to extrapolate experimental results, Corleto has recently summarized data that deals with SSTR knockout mice, which he believes could be of use for a better understanding of gastrointestinal tract functions and SST [53].

Keeping in mind the initial descriptions of SST in the CNS as well as in GI physiology, it should not surprise us that SST together with other peptides can be involved in visceral sensations such as taste [54]. In addition, homologies between taste receptors and the sequence of SST and opiate receptors have been described [55]. The relevance of this homology will be discussed in the section of heterodimers.

#### Somatostatin in nociception and its relation to mechanosensing

A series of studies have documented the relation between SST and nociception as well as with counter-regulation in inflammatory situations [56-61]. Changes in the expression of SSTR can be involved in alterations of chemical sensitivity as well as of mechanosensing in afferent mesenteric nerves [62]. Modulation of pain transmission has a complex circuitry which includes SSTR [63]. During the sensitization of nociceptors it has been demonstrated that SST interacts with the vanilloid receptor TRPV1 [64]. The family of vanilloid receptors is involved in mechanosensory conduction [65-68]. μ-Opioid receptor activation can modulate thermal hypersensitivity associated with tissue inflammation through the TRPV1 channels [69]. It is interesting to note that in experimental pulp inflammation both SST and opioid levels are found to be locally increased [70]. While these actions might seem to be unrelated to SSTR, a later section dealing with receptor dimerization will bring more light into this issue. Taking that SSTR expression is related to inflammation and nociception in the surroundings of a gastrointestinal tumor, one could expect that some of tracer binding patterns might be related to these processes.

Already in 1990, in the article by Lamberts et al. on the use of iodine-labeled octreotide [71] an anti-nociceptive action of unlabelled short-acting octreotide was described. In 1991 a similar property was described for a long-acting somatostatin analog [72]. A newer SSA, vapreotide, has also been characterized as being anti-nociceptive [73].

#### Octreotide scanning in thyroid-associated orbitopathy-what can we still learn?

In previous studies at the Medical University of Innsbruck we have been involved in the use of octreotide scanning for the evaluation of the inflammatory components of thyroid-associated orbitopathy (TAO) [74,75]. Recently, we have been able to describe musculoskeletal components in this disease based on scintigraphic data [76]. While SSTR imaging was positive in TAO patients, the use of cold Sandostatin^® ^did not fulfill the expectations of clinicians and patients. Based on the rather disappointing approaches with immune modulators for the treatment of TAO, we have recently started to apply a different diagnostic and therapeutic approach. By applying diagnostic concepts of TCM, one can characterize these patients as being Qi deficient. This clinical diagnosis coincides with experimental data from Liu et al. who used the herbal formulation Sijunzi (containing *Panax ginseng*, *Poria cocos*, *Atractylodes macrocephala*, and *Glycyrrhiza uralensis *[77]) in order to treat experimental Qi deficiency [78]. This treatment was able to lower the levels of SST in the colon mucosa.

In TAO, we have started to use a multi-agent herbal preparation based on the use of Western herbs [79]. The formulation used for TAO patients includes *Ruta graveolens *[80], *Anemone pulsatilla*, *Hypericum perforatum*, *Serenoa serrulata*, *Schisandra chinensis *[81], *Ophiopogon japonicus*, *Glycyrrhiza glabra*, and *Zingiber officinale *[79]. Hypericum, first described in 1975 [82], can affect the sub-cellular localization of the retinoid X receptor [83] and acts also as antidepressant and anti-inflammatory [84] also through interaction with the CRH-1 receptor [85]. Due to interactions of *Hypericum *with hepatic metabolism of drugs [86], it is not advisable to administer it together with other pharmaceuticals. However, an important action of *Hypericum *is that of preventing inflammation related fibrosis [87]. *Serenoa *has been mostly characterized for its use in benign prostate hypertrophy [88]. *Schisandra *can positively influence the glutathione levels and thus achieve anti-oxidative effects [89] while at the same time it protects from proteoglycan degradation [90]. The pregnane X receptor can also be activated both by *Schisandra *and *Glycyrrhiza *[91]. *Ophiopogon *has anti-inflammatory properties [92,93]. Finally, *Zingiber *can inhibit platelet aggregation and has anti-inflammatory properties [94-97]. Translating this approach into treatment terms we can describe it as a multi-agent multi-target strategy. The reader might ask now, how can this knowledge be used in Western medicine? Roth and collaborators have published several articles dealing the investigations of the receptorome, which is the portion of the proteome encoding receptors [98]. Based on this principle they have been able to identify ligands from psychoactive plants that interact with the receptorome [99]. This is a multi-agent multi-target environment of real life. Sucher has recently presented an extensive analysis of herbs for neuroprotective use which were also investigated under a multi-component multi-target approach [100]. Straube et al. have recently proposed the use of multitarget therapeutics for treating headache [101].

While the departing point of this article was to understand the characteristics of scintigraphic studies with SST analogs, we should be aware that medicinal herbs, and potentially nutrients also, can interact with peptide hormones in such a way as to increase the endogenous levels of SST [102-109]. Similar actions, i.e., raising SST levels, can also be observed for omeprazole [110], a drug which is also used in the treatment of carcinoids [111].

#### Time to think over-somatostatin is not alone-urotensin, cortistatin, and somatostatin

In 1995, two independent research groups discovered a new putative neuropeptide receptor called SENR [55,112]. This was followed by the identification of urotensin as the endogenous ligand for SENR (GPR14) [113]. Quite recently, an interaction of urotensin II and of urotensin II-related peptide with SSTR 2 and 5 has been described [114]. Recent data has also confirmed the relationship between SSTR genes and those of UII/URP which are now viewed as a super family [115]. Truncated SSTR have been recently described in rodents [116]. Neuronostatin, a peptide contained in the SST gene, has been also described quite recently [117]. New physiological relations can be expected to emerge for SST and corstistatin due to similar distribution patterns in tumors [118,119]. Another aspect of SST-cortistatin receptors is the association of the cortistatin MrgX2 receptor to nociception [120,121], thus complementing the functions of SST which were described above. Cortistatin has not only similarities with the receptor binding sequence of SST but this also applies to the SST analogs octreotide and lanreotide [122]. The initial descriptions of cortistatin were made by de Lecea et al. [123] followed by Fukusumi et al. [124]

#### The modern language of receptors: mosaics and dimers, RAMPS, and arrestins

Our medical and biochemical training has told us that one correct ligand interacts with one correct receptor. While it might be correct for *in vitro *situations where purified receptors are being used, the biological environment contains dynamic structures. Early publications on receptor cooperativity were centered on receptor systems such as the cardiac muscarinic receptors [125]. In this setting Wreggett and Welss identified receptor moieties with an apparent molecular mass of 60-75 kDa, as well as 190 and 240 kDa. These last two species were interpreted as homotrimers and homotetramers, respectively. In addition the eluted receptors were accompanied by a mixture of guanyl nucleotide-binding proteins (G-proteins) [125].

The SSTR belongs to the group of G-protein coupled receptors (GPCR). In GPCR, activation of G proteins is induced by receptor-effector coupling [126]. In 1998, Gouldson et al. presented theoretical and experimental data regarding the hypotheses of receptor dimerization based on work with the models of the beta2-adrenergic receptor [127]. They concluded that two processes were important in GPCR activation namely dimerization and domain swapping. Non-ligand receptor activation, however, can also be achieved through receptor-independent activators of G-protein signaling [128].

Another nomenclature for GPCR is that of the seven-transmembrane helical receptors (7TM). Several articles have described characteristics of this family of receptors [126,129,130]. The five main types of families can be summarized by the term GRAFS which includes Glutamate, Rhodopsin, Adhesion, Frizzled/taste2, and Secretin receptors [131,132]. GPCR have the property of forming dimers, either homo- or heterodimers [133,134]. This type of association has been also termed receptor mosaics [135-139]. Keeping these facts in mind, the reader of this review was already introduced to the concept of fractal analysis of ligand-receptor interactions [13]. These ideas have been already included in modern models that look at ligand binding in the "age of dimers" as we should acknowledge [140,141]. Further steps in these models are the evaluation of dimer symmetry [142] as well as the structural form of these receptor mosaics which is important for signaling, trafficking, and oligomer intercommunication [143]. Besides these GPCR models, structural genomics have been used for protein expression, purification, and crystallography [144].

In the field of SSTR, the year 2000 marked the starting point for new knowledge regarding dimer formation. Rocheville et al. described the formation of functional homo- and hetero-dimers [145]. This experiment unveiled novel biochemical properties of the ligand-receptor interaction in the sense of molecular cross-talk among the receptor subtypes. Hukovic et al. have shown an agonist-dependent regulation of cloned human SSTR1 [146]. *In vivo *studies have shown a positive effect of pre-exposure of SST on the expression of its own subtype 2 receptors in the arcuate nucleus [147]. In the following years, more information has been gathered. Differences in the dimerization of SSTR subtypes have been investigated by the research groups of Pfeiffer, Grant, and Jacobs [148-150]. These studies revealed differences in receptor kinetics depending on the type of dimers. Durán-Prado and collaborators have recently summarized data on heterodimer formation in relation to SST signaling and control [151]. Besides the situation of dimerization induced by an agonist, Reubi et al. have recently described the effect of a DOTA chelator that changes the action profile of the tracer, i.e., from antagonist to agonist [152].

While we are accustomed to think exclusively one way on SST and SSTR while looking at SSTR imaging, real-life biochemistry might be different. Besides the property of SSTR dimer formation, heterodimers involving other receptors types are now known or have been developed experimentally. One type of heterodimer is related to dopamine and SSTR which was originally described by Rocheville [153]. These hetero-oligomers of dopamine and SSTR had enhanced functional activity. The second type of heterodimer includes opioid and SSTR, which were originally described by Pfeiffer et al. [154,155]. In this context, it is important to mention other work done on opioid receptors. Using an *in vitro *system Gomes et al. [156] have observed that rather low doses of some delta-selective ligands can lead to a significant increase in the binding of a mu receptor agonist. It is important to keep in mind these heterologous interactions since patients with NET might be treated at some time with these types of pharmacological agents. Analysis of the GPRC genome shows interesting relationships of the SST and opioid receptors [157]. The MCHR2 and NPBWR2 genes are found at the roots of the SST and opioid receptors branch. GPR32 and GPR33 are under the SST and opioid receptor cluster. Among opioid receptors heterodimers of mu and delta receptors can be found [156]. A physiological meaning of this type of heterodimers might be related to nociception.

New data on receptor functioning requires also a new language. Taking an example of receptor dimerization in relation to SST [153] Kenakin [158] describes new interactions on the SSTR5-D2 heterodimer, termed conduit, having SST 14 as a ligand, termed guest. The dopamine receptor D2 agonist quinpirole increases the binding affinity of somatostatin-14 while the dopamine receptor D2 antagonist sulpiride decreases the binding. These last two are called modulators. O'Toole et al. have described the co expression of SSTR2 and D2 receptors in GEP tumors [159]. The authors concluded that bi-specific agonists such as SST(2)/SST(5) or SST(2)/D(2) could be tested in these tumors. This type of reasoning has stimulated research work on the side of the ligands leading to the synthesis of ligands such as BIM23A357 and BIM23A770 which can bind both SST and dopamine receptors [160]. Recently, Arvigo et al. [161] have described somatostatin and dopamine receptor interactions in cell lines (prostate and lung cancer). Synergistic stimulation had effects on the inhibition of cell proliferation.

Following ligand binding on the cell membrane, further mechanisms have to control the signaling of the ligands. One of these mechanisms involves arrestins. Receptors can be classified according to their ability to bind arrestin [162]: "Class A receptors (ß2 adrenergic receptor, mu opioid receptor, endothelin type A receptor, dopamine D1A receptor, and α 1b adrenergic receptor) can bind ß-arrestin2 with higher affinity than ß-arrestin1 and do not interact with visual arrestin. In contrast, class B receptors (angiotensin II type 1A receptor, neurotensin receptor 1, vasopressin V2 receptor, thyrotropin-releasing hormone receptor, and substance P receptor) bound both ß arrestin isoforms with similar high affinities and also interacted with visual arrestin" [162]. The arrestins, ß-1 and ß-2, are negative regulators of GPCR signaling which translocate to the cell membrane. Here, they bind the occupied receptors. This is followed by uncoupling of the receptors from G-proteins, leading finally to internalization, and by this, desensitation occurs [163]. Further actions of arrestin on histone acetylation and gene transcription have been described [164]. In a similar way as it happens with GPCR, the arrestins can also dimerize [165]. SSTR regulation by arrestins has also been demonstrated in recent years [166-168]. Finally the so-called RAMPS function as accessory proteins that are needed for the adequate placing and function of certain GPCRs [169].

#### GPCR, caveolae, lipid rafts, and oxidative stress

The cellular localization of the receptors implies that they have to interact with the membrane and this is a function that depends on its physical properties. I will mention few structures that are relevant in this context. The caveolae membrane system describes a functional complex related to the delivery of molecules to specific locations in the cell [170]. Subunits of G proteins can bind to caveolae so that their function is also related to these elements [171-173]. Finally, the structure of a receptor is connected to cholesterol [174-176]. A reduction of cholesterol can lead to an increase in ligand binding, however, the level of intracellular signaling might be reduced [177]. Not only cholesterol (and maybe cholesterol modifying therapies) but also micronutrients can play a role on membrane fluidity [178]. Membrane rigidity depends also on lipid peroxidation [179]. Receptor density and membrane fluidity can be influenced by oxidative stress [180,181]. While oxidative stress can be sought within the diseased organ, one should also consider potential side effects of medical actions. We have recently described the negative influence of radiation exposure during peptide receptor radionuclide therapy on Se levels [182]. By decreasing Se levels, several protective selenoproteins will be compromised resulting in impaired protection against oxidative stress [183]. It follows that nutrition, anti-oxidants, and lipid-modifying therapies have to be included in our vision of receptor function [184] and possibly modulation. Among the caveolins [185], Caveolin-1 is currently being investigated in the context of tumor development [186]. Regulating mechanisms that maintain its expression could turn to be a potential tumor regulator due to tumor suppressor functions.

#### The total environment in the light of PET/CT imaging-images and postulates

**Figure 1:**
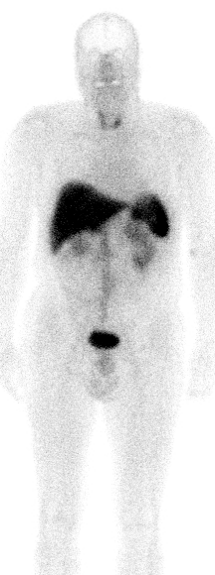
**99mTc^99^-HYNIC-TOC uptake in fibrotic scar tissue after median laparotomy**.

**Figure 2:**
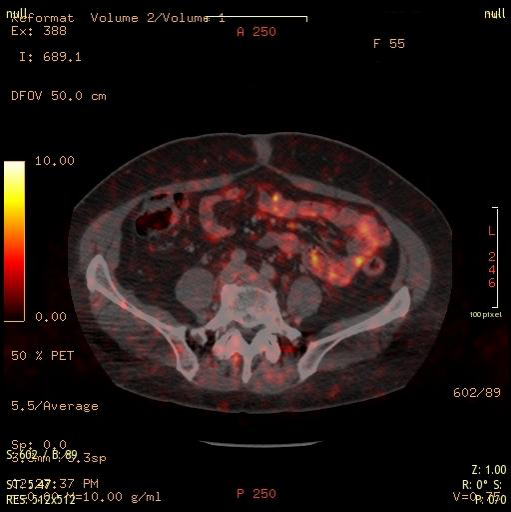
**PET/CT Ga^68^-TOC uptake in intestinal structures, in abdominal muscles, and abdominal fatty tissue**. The image was taken from a 56-year-old female patient with a NET.

When we carry out SSTR imaging for NET diagnosis, we are conditioned *a priori *to consider octreotide uptake as tumor expression. We should realize that SST is only one player among others in a complex system [187-192]. When we do SSTR imaging, we have to realize that modern imaging techniques have the potential of delivering new evidence on the distribution of SSTR. Based on the use of modern PET/CT imaging with a Ga^68^-labeled octreotide tracer, it is now possible to detect tracer uptake in bodily structures that have not been considered before. Figure 1 presents the uptake of 99mTc-labeled HYNIC-TOC in a fibrotic abdominal surgical scar, showing that tissue repair involves SSTR. An "ignored" finding in NET patients can be seen in Figure 2 where tracer uptake within a small intestinal loop is demonstrated. On theoretical grounds, one can suspect that an inflammatory process is present. Inflammatory gut diseases and NET have been discussed above.

**Figure 3:**
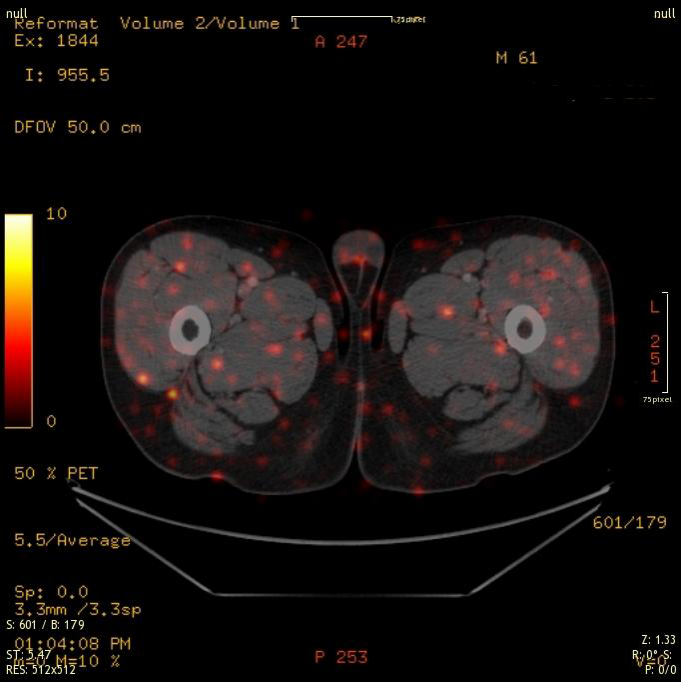
**A 61-year-old male patient presenting intense uptake in muscular structures and in fatty tissues**. Low levels of Se were documented in this case.

**Figure 4:**
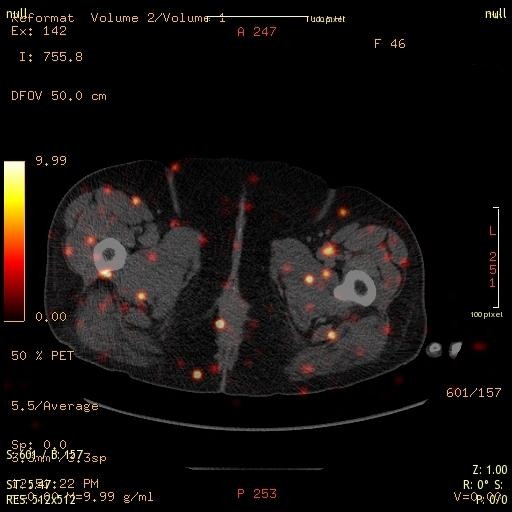
**A 46-year-old female patient with axial mis-alignment, the mid-line is shifted to the left**. The uptake intensity is greater as in the previous case. Axial mis-alignment could influence mechanotension of the muscular structures and produce enhanced SSTR expression.

Figures 3 and 4 depict octreotide localized uptake in peri-muscular structures of the thigh both in a male and a female patient. The images have been taken from PET/CT studies of patients with NET investigated with 68Ga-DOTA-TOC. The distribution pattern delineates muscular fasciae [193]. For a general description of the distribution of superficial and deep fasciae of the body, the readers are referred to Gerlach [194]. Such fascia structures have a longitudinal distribution between the muscles and are considered to be involved in epimuscular myofascial force transmission (e.g., Figure nine in [195]). Conventional three-view reconstruction algorithms in Nuclear Medicine do not produce a suitable image of this type of aligned structures. An adequate approach to achieve this would be to apply the principles of diffusion spectrum MRI tractography [196]. The applicability of this method in the investigation of myocardial, i.e., muscular structures, has been recently demonstrated [197,198].

Between the fasciae and connective tissue in the body, tensile forces are active. Standerwick and Roberts have described these relations for craniofacial growth [199]. I propose that the punctual uptake is in relation to mechanosensing, since distension of the muscles, e.g., through eccentric exercise, will act on these structures [200-202]. This is the basic assumption that we have included in the musculoskeletal model of thyroid-associated orbitopathy [76].

The careful observer of figures will also recognize tracer uptake in the subcutaneous fatty structures. While it is not possible to deliver an exact anatomical correlate of this binding site, the distribution pattern might be involved in processes of metabolic signaling such as lipolysis [203,204]. In humans, changes of both SST secretion and SSTR expression has been described in conditions of infection and inflammation of adipose tissue [205].

#### Recent data on SSTR in the early years of the new century

In the process of editing the final version of this manuscript, I came across some recent data on SSTR which should be mentioned here. The data presented above has in a way a historical character. New developments, however, change the face of science.

One of the most relevant aspects on SSTR is that of truncated somatostatin receptors. The group of Córdoba-Chacón et al. have described a series of new splicing variants of the SSTR-5 molecule in pituitary tumors as well as in rodents [206,207]. The result of these alterations is the appearance of SSTR that have different numbers of trans-membrane domains (TMD). In their recent publication [208], they describe some characteristics of the human spliced variants of the SST receptor subtype 5. Among these is the novel evidence that demonstrate that an SST can react in different ways to very similar analogs of SST. One clinically important issue is the finding of SST analog-resistant somatotropinomas where the variant called hsst5TMD4 was found [209]. In their review on the topic of truncated somatostatin receptors, Córdoba-Chacón et al. discuss the issues that led to a novel reasoning for the interpretation of SST-knockout models [208]. The data discussed originated new explanations for the different effects of SST and cortistatin at different levels. This finding is of outmost importance in the clinical management of patients with NET, since sole tracer binding will not be able to identify the existence of this truncated receptor variant, while at the same time therapeutic efforts with either unlabelled or labeled SSTA might remain unsuccessful. Implementation of these methods in cases of NET is badly needed!

Besides changes in the receptor peptide structure, a further factor that influences binding is the carbohydrate component of the moiety [210]. In a recent article, Møller et al. discuss the influence of the carbohydrate component on SSTR (section 2.3.2. in [211]). Glycosylation moieties are present in SSTR5 [212].

An ample description and discussion on somatostatin receptors based on the experience with patients with acromegaly has been recently presented by Colao et al. [213]. The clear advantage in the field of acromegaly it that it is possible to investigate the efficacy of different therapeutic approaches by determining the targets, e.g., growth hormone, IGF-I, as well as the characteristics of the pituitary tumor. This is not always the case in cases with NET since the decision to initiate treatment might simply come from scintigraphic results [214] even when the patients have no significant endocrinological alterations. One important aspect in Colao's paper is the description of SSA resistant cases. This situation might also occur in NET.

Man is not alone in nature. Developmental aspects of receptors have been presented by other authors. Gahete et al. have summarized data related to the development of somatostatin receptors from fish to mammals [215]. Emphasis has been put on the section on new SST receptors. In a similar way Vaudry et al. have described the evolution of urotensin receptors [216]. These early forms of SSTRs have the potential of being more easily accessed for scientific research.

#### Conclusions: the fiction in science [217,218]

Science is an art. In doing medical research, we attempt to recognize the elements involved in this art frame and await confirmation of hypotheses by going through empirical and evidence-based paths [219-226]. In view of the biochemical complexity of receptor interaction outlined in this review, I believe that the most suitable graphical and operational representation is that of coalition chess as proposed by Arnold Schoenberg in the 1920s [227]. Coalition chess involves four players with each player moving different chess figures. It allows the possibility to form coalitions between the players-as agonists or antagonists. Using this chess variant as a concept, one can imagine "the players" being involved in receptor interaction. Modern methods like functional proteomics and genomics [228], functional nutrigenomics [229], receptorome mining [98], chemogenomics [230], and metabolomics [231] will surely gain relevance in the field of Nuclear Medicine in order to decipher these multiple interactions. Multiagent multitarget procedures, similar to the herbal combination described herein, could be analyzed by nutriome methods [232]. For NM, one could envision the use of multivalent tracers or tracer mixtures in therapeutic situations. In addition, we can expect that a new terminology will be proposed for receptor forms and interactions [233,234]. The influence of morphogens and morphostats on NET is also added as a research direction to be kept in mind [190,191,235-240]. Finally, basic research will start to get involved with spliceosome dynamics [241] in order to provide answers to the truncated forms of SSTR.

**Figure 5:**
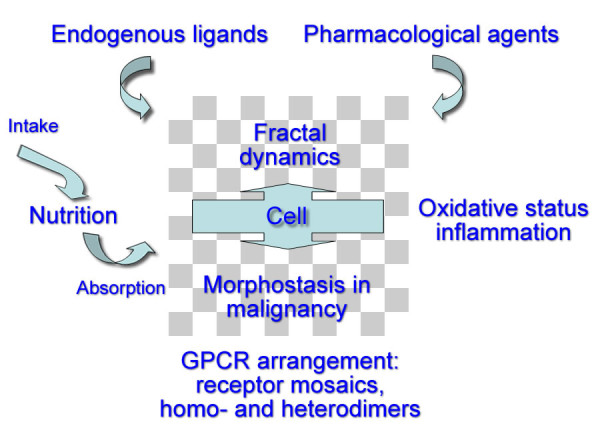
**Graphical summary of this review**. This graphical summary of this review presents several real-time players that make up the total environment behind octreotide scintigraphy. (1) Nutrition: sufficient macro- and micronutrients of good quality are the starting point. Intestinal function will have an influence on absorption. (2) Endogenous ligands: somatostatin, corticostatin, urotensin, neuronostatin. (3) Pharmacological agents: octreotide, DOTA-chelators in tracers, lanreotide, omeprazole, lipid modifiers. (4) Ability to cope with oxidative status and inflammatory conditions. (5) Characteristics of the GPCR system: homo- and heterodimers, truncated receptors, RAMPS, arrestins, etc. (6) Morphostats, and cell regulators in malignancy.

I present a graphical summary of this review in Figure 5. Upon seeing the figure, the reader might recall the words of William Wordsworth: "My heart leaps up when I behold a rainbow in the sky". I propose that when we look at octreotide scintigraphy, while still strictly sticking to the procedure guidelines [242,243], we should think on inflammation, nociception, mechanosensing, chemosensing, fibrosis, taste, vascularity, and also tumor. An increase of any of these single processes might result in increased tracer uptake making up a rainbow in our imaging sky. While people tend to believe what they see, and Nuclear Medicine is an imaging specialty indeed, we should not forget the basic principle of uncertainty in science [244]. Quoting Castillo while he refers to Feynman [245] one reads: "He goes on to say that if we are free of doubt and ignorance, we will not get any new ideas and make no progress".

## Abbreviations

68Ga-TOC: ^68^Ga-DOTA^0^-Tyr^3 ^octreotide; 7TM: seven7 -transmembrane helical receptors; CNS: central nervous system; GI: gastrointestinal; GPCR: G-protein coupled receptors; MRI: magnetic resonance imaging; NET: neuroendocrine tumors; RAMPS: receptor-activity-modifying proteins; Se: selenium; SSA: somatostatin analogs; SST: somatostatin; SSTR: somatostatin receptors; TAO: thyroid- associated orbitopathy; TCM: traditional Chinese medicine; TMD: transmembrane domains.

## Competing interests

The author declares that he has no competing interests.

## Authors' contributions

RM conceived the idea, wrote the manuscript, and drew the graphics.

## Author information

Roy Moncayo is trained in Internal Medicine, Endocrinology, Nuclear Medicine, Chinese Acupunture, and Western Herbal Therapies and holds a MAS in Health and Fitness. He is Deputy Head of the Department of Nuclear Medicine at the Medical University in Innsbruck. His clinical experience with octreotide is centered on thyroid-associated orbitopathy. He carries out complementary work on musculoskeletal disorders at WOMED, Innsbruck.
